# Septic arthritis of the temporomandibular joint–a case report and review of the literature

**DOI:** 10.3389/froh.2024.1496094

**Published:** 2025-01-07

**Authors:** Shareef Araidy, Naseem Maalouf, Eran Front, Imad Abu El-Naaj

**Affiliations:** ^1^ Department of Oral and Maxillofacial Surgery, Tzafon Medical Center, Affiliated with Azrieli Faculty of Medicine, Bar Ilan University, Ramat Gan, Israel; ^2^ Unit of Periodontology, Tzafon Medical Center, Affiliated with Azrieli Faculty of Medicine, Bar Ilan University, Ramat Gan, Israel; ^3^ Department of Oral and Maxillofacial Surgery, Tzafon Medical Center, Associate Professor at the Azrieli Faculty of Medicine, Bar-llan Univesity, Ramat Gan, Israel

**Keywords:** temporomandibular joint (TMJ), diagnosis, septic arthritis, complications, temporomandibular disorders (TMDs)

## Abstract

Septic arthritis occurring in the temporomandibular joint (TMJ) has received significantly less attention than it deserves. This condition can severely compromise joint functionality, especially if left untreated. Its typical presentation includes pain, fever, swelling, and the loss of TMJ functions. We report a case of TMJ septic arthritis arising in the left joint of a 52-year-old woman. Our report, along with a review of the literature, highlights the importance for early detection of TMJ septic arthritis, its clinical and radiographic characteristics, as well as the management of this rare disease and the proposed etiologies of its pathogenesis. Raising awareness among healthcare providers can promote early diagnosis, improving outcomes and preventing complications of TMJ septic arthritis.

## Introduction

The temporomandibular joint (TMJ) is a bilateral synovial articulation in the craniofacial region and plays a key role in important functions, such as chewing, swallowing, and speaking (1). Specifically, the TMJ serves as the hinge joint that connects the mandibular condyle and acts as the articular surface to the glenoid fossa of the temporal bone (1).

Septic arthritis of the TMJ is a rare condition, with fewer than 100 cases documented in the literature (2, 3). Although typically linked to *Staphylococcus aureus*, it can also be caused by various other microorganisms (4). The infection often arises through hematogenous dissemination, local spread, autoimmune disorders, blunt trauma, or surgical interventions (4). This condition can affect both children and older patients. However, it is more commonly seen in older individuals with underlying comorbidities that render them immunocompromised, with symptoms often presenting rapidly (5). The symptoms of TMJ septic arthritis include limited mobility of the affected joints, preauricular edema, headaches, fever, and general body weakness (2, 3, 4).

This article presents a rare case of septic arthritis of the TMJ in a 52-year-old female, highlighting the challenges in diagnosing and treating this unusual condition. Additionally, we present a literature review, offering insights into the condition's etiology, diagnosis, and management.

## Case report

This report presents a 52-year-old female with a history of hip and knee replacements due to osteoarthritis, as well as a hysterectomy. She presented to the emergency department (ED) with severe facial and ear pain, swelling, tinnitus, and difficulty opening her mouth. These symptoms developed three days prior to her arrival at the ED. Notably, the patient had no prior history of temporomandibular joint disorder (TMD), confirmed by a review of her medical records.

We initiated the diagnostic process with a clinical examination that included both extra-oral and intra-oral assessments. Extra-oral examination revealed left pre-auricular swelling, accompanied by tender, local warmth and redness. Her mouth opening was restricted due to pain. Following the administration of analgesics, she achieved a full range of mouth opening with no audible joint clicks or crepitus. Muscular palpation elicited tenderness in the left masseter and temporalis muscles. The intra-oral examination uncovered an unbalanced occlusion with a left-side open bite, several carious lesions of the upper teeth without signs of acute dentoalveolar infection, and no further pathology of the tongue or floor of the mouth.

Laboratory tests conducted upon her arrival showed leukocytosis and elevated C-reactive protein (CRP) levels.

**Figure 1:**
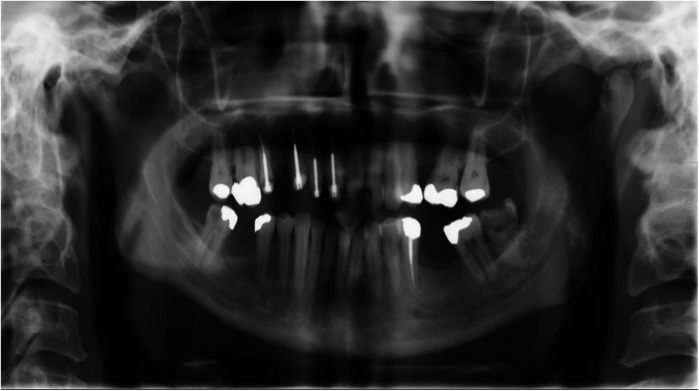
Panoramic radiogram taken upon admission to the emergency department. The left condyle appeares more pronounced compared to the right, showing an enlarged intra-articular space.

**Figure 2:**
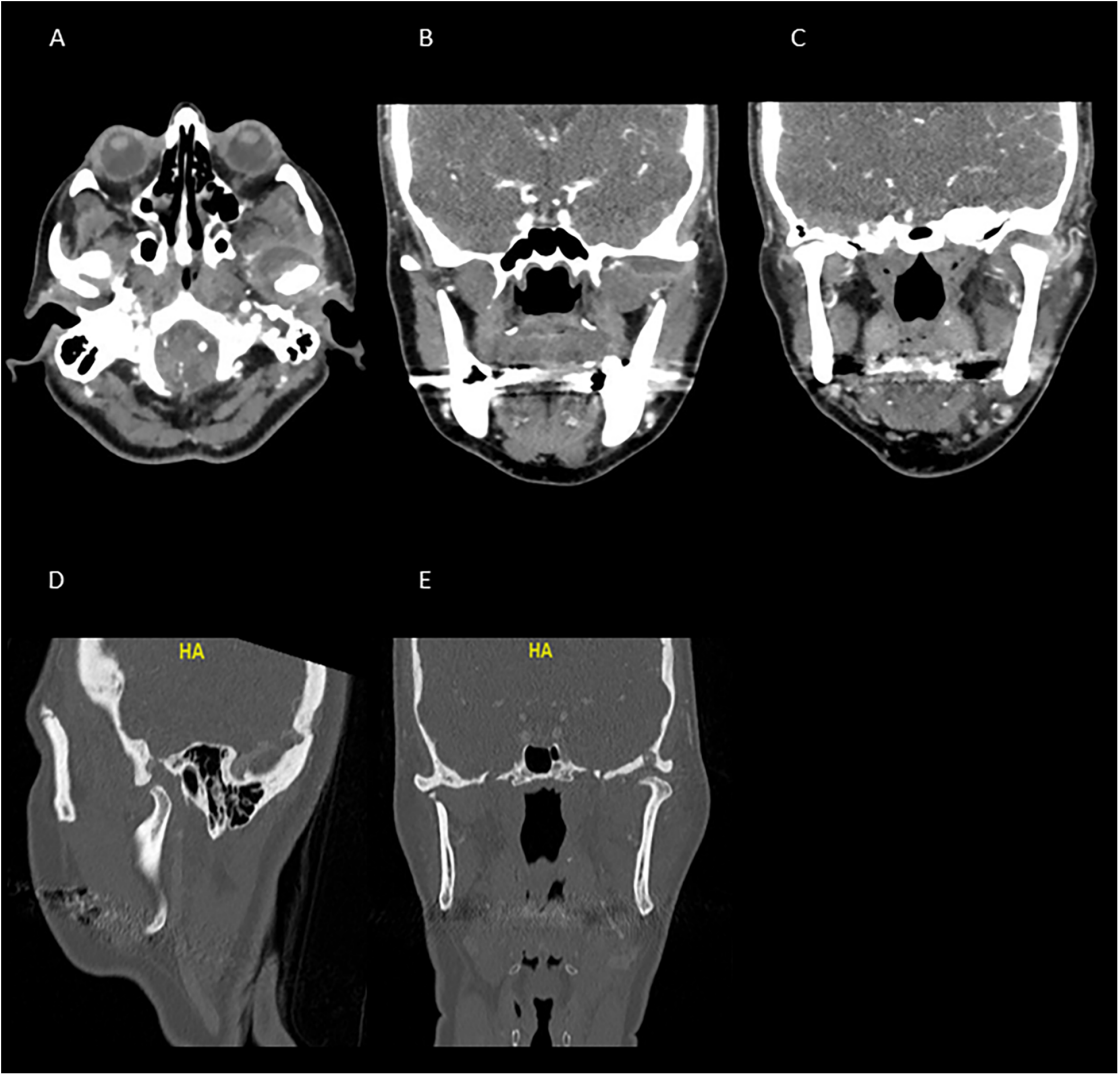
CT scan taken upon arrival to the emergency department. The images **(A–C)** show a significant hypodense area at the anterior part of the left TMJ, demonstrating signs of inflammation, edema, and collection around the left joint. **(A)** Axial view, **(B,C)** coronal view. The images **(D,E)**, demonstrate an erosion of the glenoid fossa and the significant bone involvement, suggesting the presence of an advanced pathological condition. Images **(D,E)** show the sagittal and coronal views, respectively, at the bone window.

**Figure 3:**
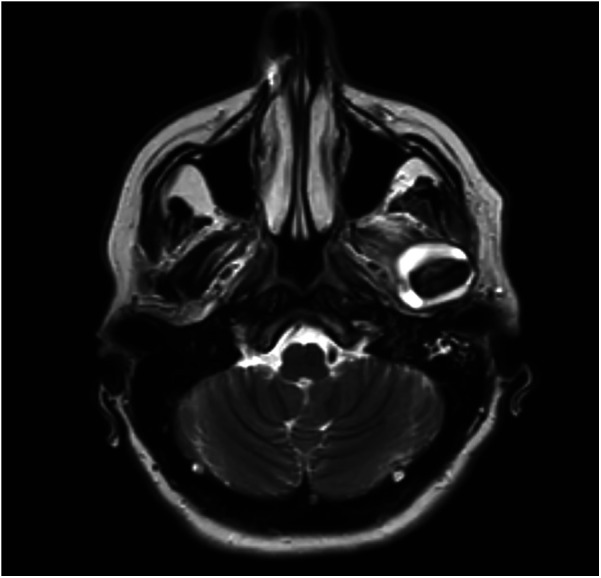
T2 sequence MRI of the TMJ, coronal images, revealing hyperintensity and joint effusion on the left joint.

We subsequently conducted radiographic examinations. A panoramic radiogram demonstrated asymmetry in the condylar area. The left condyle was more prominent than the right counterpart, with increased intra-articular space (Figure 1). A computed tomography (CT) scan revealed features indicative of soft tissue involvement and inflammatory changes, including the presence of a large subperiosteal abscess on the anterior part of the left mandibular condyle (Figures 2A–C). Notably, there was an absence of osteoarthritic radiographic changes in the left condyle (Figures 2A–C). Additionally, the detection of a partial cortical fracture and elevated periosteum in the middle cranial fossa further underscored the severity and extent of bone involvement (Figures 2D,E), indicating that the condition was in an advanced stage. Magnetic resonance imaging (MRI) pinpointed a hyperintense signal, indicating an edema and inflammation in the left joint (Figure 3).

All the clinical and radiographic findings confirmed the diagnosis of severe infectious condition of the left TMJ. This necessitated immediate treatment and close monitoring. Therefore, we initiated therapy by administering empirical antibiotics, specifically Augmentin {Amoxicillin 875 mg + Clavulanic Acid 125 mg [GlaxoSmithKline (GSK), Portugal]}. It was administered intravenously (IV) for three days, at a dose of one gram thrice daily.

During the admission period, a multidisciplinary medical team assessed the patient. The neurological examination revealed the presence of autoinflammation at the base of the skull. While the autoinflammation showed no signs of meningeal involvement, it still affected the adjacent dura. An ear, nose, and throat (ENT) consultation ruled out any otological pathology, noting that the patient's middle ear and mastoid structures appeared well-ventilated. Furthermore, considering the patient's history of arthritis, which included a total knee replacement, right hip surgery, and left knee arthroscopy, a rheumatologic evaluation was warranted. This led to a series of investigations, including clinical and radiological assessments (x-rays of the pelvis, hips, and hands) as well as laboratory tests, such as rheumatoid factor and serum uric acid levels. Ultimately, none of these revealed evidence of an acute systemic arthritic process.

To further investigate the advanced infectious process, the patient was subjected to decompression and aspiration procedures on the left joint. The extracted fluid was analyzed using a pan-bacterial polymerase chain reaction (PCR) technique and cytological assessment. The cytology report highlighted the presence of histiocytes and leukocytes, predominantly neutrophils, without detecting any cancerous cells. However, the PCR analysis yielded negative findings for an identifiable pathogen.

So far, we suspected a pathological condition affecting the TMJ, prompting a broad differential diagnosis. Given the patient's history of arthritis, TMJ osteoarthritis may present with overlapping symptoms, making it a plausible diagnosis. Additionally, systemic manifestations of arthritis, such as fever and malaise, can resemble those observed in autoimmune conditions like rheumatoid arthritis. Other differential diagnoses to consider included septic arthritis, adjacent soft tissue cellulitis, gout, pseudogout, traumatic effusions, and synovial chondromatosis.

However, based on the patient's clinical presentation, which included the acute onset of joint pain, pre-auricular swelling, tinnitus, trismus, and ipsilateral open bite—along with no prior history of TMD or blunt trauma—there was significant evidence of an acute pathological joint condition. Furthermore, ENT, neurological, and rheumatological evaluations, along with laboratory findings, supported the diagnosis of an acute infection. Imaging results revealed a large subperiosteal abscess on the anterior part of the left mandibular condyle, reinforcing this suspicion.

Taken together, the clinical, radiological, and laboratory findings raised a presumption for septic arthritis. Despite negative microbiological cultures, our suspicion remained high for septic arthritis, as existing literature demonstrated that failure to identify a specific pathogen was relatively common in septic arthritis (2).

After completing the initial course of treatment and observing minimal improvement in clinical outcomes, a second treatment regimen was implemented based on the recommendations of infectious disease experts and the results of the culture test. The patient started taking an oral dose of Flagyl [Metronidazole (Pfizer, United States)] 250 mg, three times a day, as well as IV Rocephin [Ceftriaxone (Roche, Switzerland)] one gram, once a day. The latter antibiotic regimen resulted in marked improvements in mouth opening, edema, and pain. This positive clinical response was further supported by a reduction in CRP levels, which decreased from 9.14 mg/dl to 0.76 mg/dl within one week following the aspiration procedure and the antibiotic therapy. Similarly, a significant reduction in her leukocyte count was observed, which decreased from 14.2 10^3^ /µl to 5.3 10^3^ /µl.

**Figure 4:**
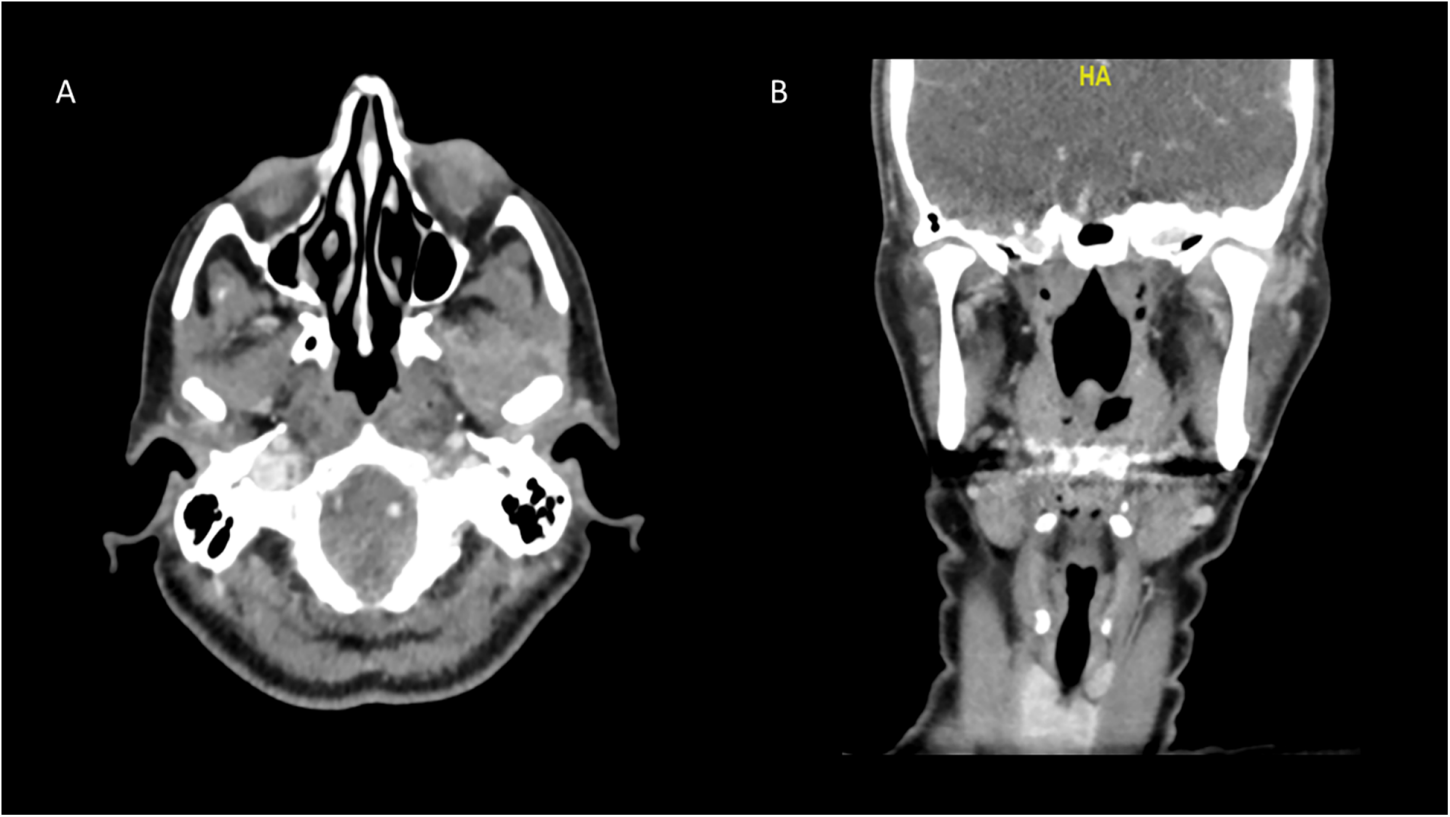
CT scan two weeks after administration of antibiotics, demonstrating almost total resolution of the collection on the left joint. This notable reduction in the collection demonstrates a positive response to treatment and indicates effective management of this severe condition. **(A)** Axial view, **(B)** coronal view.

Two weeks after the antibiotic therapy, the patient showed even more notable clinical improvement. This included a significant reduction in preauricular edema, with only slight residual swelling, as supported by the radiographic examination (Figure 4), with no signs of redness or local warmth. Spontaneous mouth opening improved markedly, and intraoral examinations revealed better occlusal relations. This favorable response to antibiotic therapy reinforced our hypothesis of TMJ infection. Upon discharge from the hospital, the patient was prescribed a course of antibiotics (Rocephin 1 gram once a day and Flagyl 250 mg three times a day) for an additional four weeks. She attended weekly follow-up appointments for six months to monitor her healing. In addition to her medication regimen, she was advised to participate in supportive treatments, such as physical therapy and exercises aimed at improving jaw mobility and strength. During the initial follow-up visits, she reported fatigue, ongoing pain, and slightly limited mouth opening. This contributed to her overall discomfort and restricted her daily activities. However, after several visits, she began to show noticeable improvements in her overall condition, including a reduction in pain levels, enhanced TMJ mobility, and a gradual restoration of her mouth opening.

## Discussion

Septic arthritis of the TMJ is a rare, life-threatening condition caused by a pathogenic infection (2). While the incidence of TMJ septic arthritis is relatively low, with limited cases reported in the literature (2), it remains crucial to understand the disease and diagnose the condition due to its potential to arise from systemic or localized bacterial infection in the joint's physiology. While this disease can affect individuals at any age, it is considerably more common among adults compared to children (5). The risk factors for contracting septic arthritis include previous joint surgeries, immunocompromised patients, trauma, and systemic conditions that can facilitate the disease (6).

The high vascularity of the synovium, combined with the absence of a basement membrane, significantly increases the likelihood of the TMJ being exposed to pathogens, with hematogenous spread serving as the primary route of transmission to this area (3). Among the various pathogens, bacterial infections are often the leading cause of TMJ septic arthritis, with *Staphylococcus aureus* identified as the predominant microbe (7, 8). Other bacteria, including *Pseudomonas aeruginosa*, *Streptococcus* species, *Haemophilus influenzae*, particularly in association with dental infections or injuries, may also be involved in the etiology of septic arthritis. At times, the process of infection may be facilitated by fungal or viral agents as well (9).

Patients with TMJ septic arthritis typically present with a sudden onset of pain, swelling, and restricted jaw mobility (4), which impair essential functions such as chewing and speaking, along with additional systemic clinical manifestations that may include fever, general malaise, and weakness (4). Extraoral signs often include swelling, redness, and joint tenderness, trismus, deflection of the mandible to the side upon opening, and movement-evoked pain on palpation (4). Intraoral examinations show changes in articulation, including an open bite of the affected side (4). Some other symptoms associated with TMJ septic arthritis may overlap with those of other TMDs, complicating the diagnostic process (10). For instance, TMJ osteoarthritis may exhibit a similar clinical presentation; however, it typically lacks the acute inflammatory signs characteristic of septic arthritis. Furthermore, the generalized manifestations of septic arthritis, such as fever and malaise, can resemble those found in rheumatoid conditions (11). Other differential diagnoses to consider include common TMDs, adjacent soft tissue cellulitis, malignant otitis externa, gout, pseudogout, rheumatologic diseases, traumatic effusions, parotid tumors, neoplasms like ganglion cysts, pigmented villonodular synovitis (PVNS), and synovial chondromatosis (12).

Consequently, the diagnostic approach for TMJ septic arthritis should encompass a comprehensive assessment, starting with a thorough patient history and physical examination. Advanced imaging techniques, such as MRI, facilitate visualization of the synovial lining and adjacent soft tissues, enabling identification of effusions, bone marrow edema, and other signs of infection (13). Additionally, CT scans are utilized to detect bone malformations and abscess formation. If clinical and imaging results suggest septic arthritis, synovial fluid aspiration should be performed to confirm infection (13).

Early diagnosis and appropriate treatment are vital to the prognosis of TMJ septic arthritis (7, 14). Following treatment, continuous monitoring is necessary to identify possible recurrences, residual symptoms, or complications (15). Healthcare providers must remain vigilant in recognizing TMJ septic arthritis, particularly in immunocompromised individuals and patients with a history of trauma, as these factors heighten the risk of delayed diagnosis and treatment (16). Multidisciplinary collaboration involving orofacial surgeons, rheumatologists, and infectious disease specialists is essential to facilitate rapid and accurate diagnosis, optimizing patient outcomes.

Untreated TMJ septic arthritis can lead to severe complications, including joint destruction and ankylosis, where the articulating surfaces of the joint fuse, causing permanent restrictions in jaw movement (4). This condition can persist despite medical interventions and severely diminish the patient's quality of life. Additionally, septic arthritis of the TMJ can result in cellulitis or abscesses in surrounding tissues, leading to pain, swelling, and inflammation, which may necessitate drainage or surgical intervention.

If the infection spreads, it can lead to sepsis, increasing the risk of systemic inflammation and potential organ failure (17). The infection may also penetrate deeper tissues, causing osteomyelitis or even dural involvement, further complicating the prognosis (17). Early detection through CT and MRI scans is essential for identifying bone inflammation or degeneration, thus preventing severe complications. Prompt intervention is crucial to manage the infection effectively and reduce the risk of long-term neurological sequelae (17).

Recent advancements in biological therapies for septic arthritis have introduced innovative treatment options that provide more targeted and effective approaches for managing this condition (18). Among the most promising therapies currently under investigation are tumor necrosis factor-alpha (TNF-α) inhibitors, which function by blocking TNF-α, a critical cytokine in the inflammatory response (19). By selectively inhibiting TNF-α activity within the joint space, the inflammatory process is significantly reduced (19).

This article aims to present an extraordinary case of septic arthritis affecting the TMJ, a rare and frequently under-recognized disease that contributes to the limited number of documented cases in the literature. In Supplementary Table S1, we provide a review of all cases referenced in this article, representing the currently reported cases in the literature to the best of our knowledge. Additionally, we provide a comprehensive review of the clinical presentation, diagnosis, and management of this disease, aiming to enhance awareness and understanding among healthcare providers.

While this study provides valuable information on septic arthritis of the TMJ, it is essential to acknowledge its limitations. One significant limitation is that the PCR results were negative and failed to identify a specific pathogen. The exact cause of the negative culture results remains uncertain. However, we propose that a likely explanation lies in the timing of the joint aspiration and fluid analysis, which were performed a few days after the initiation of the antibiotic regimen. This delay may reduce the likelihood of isolating the causative organism, as noted by Al-Khalisy et al. (4). Such limitations hinder the accurate identification of the causative organism, complicating both the diagnosis and management of the condition. Although joint aspiration and culture are frequently employed in clinical practice, negative results are relatively common, as demonstrated in previous review (2). Therefore, a combination of thorough patient history, physical examination, laboratory findings, and imaging is required to achieve a definitive diagnosis.

## Conclusion

In summary, septic arthritis of the TMJ is a rare and often under-recognized condition that poses significant diagnostic and management challenges. This case report illustrates the complexity involved in diagnosing septic arthritis and highlights the importance of a thorough clinical assessment, combined with laboratory tests and advanced imaging techniques. The successful treatment of our patient demonstrates that effective management can be achieved through multidisciplinary collaboration and personalized treatment strategies, emphasizing the integration of clinical expertise with patient-specific care. By increasing awareness of this condition among healthcare providers, we can facilitate earlier diagnosis and intervention, ultimately improving patient outcomes and preventing potential complications associated with untreated septic arthritis of the TMJ. Continued research into the pathophysiology and management strategies for this condition is essential to further improve patient care and outcomes.

## Data Availability

The original contributions presented in the study are included in the article/Supplementary Material, further inquiries can be directed to the corresponding author.
