# Supplementary material for: Septic arthritis of the temporomandibular joint–a case report and review of the literature
Source: Front Oral Health. 2025 Jan 7;5:1496094. doi: 10.3389/froh.2024.1496094 (PMC11756522; doi:10.3389/froh.2024.1496094)
Supplement: Supplementary file 1 [file Datasheet1.pdf]

Summary of TMJ septic arthritis cases referenced in this article

Author / Year	Cases (number)	Age/Sex	Presentation	Diagnosis	Treatment	Outcome
Omiunu et al., 2021	93	35.7/NA	Appearance of trismus, pain at the jaws, and periarticular swelling	Radiograph, CT scan, MRI, ultrasound	Surgeries, antibiotics	Faster evaluation of the condition, and treat with a wide range of antibiotics. Surgery can save lives when the condition is severe
Jovanović et al., 2022	91	Adults	Pain, presence of trismus, infrequent fever	Isolating the streptococci and staphylococcus aureus	Administration of metronidazole and third-generation cephalosporin	Required administering antibiotics for a longer duration, significant need for surgical measures for its management
Al-Khalisy et al., 2015	1	24/M	Erythema fever, general jaw pain, restricted mouth opening and swelling on the	CT , MRI scan and Isolating staphylococcus aureus	Antibiotics and drainage of effusion	Complete recovery

Summary of TMJ septic arthritis cases referenced in this article

			periauricular area			
Dias Ferraz A et al., 2021	7	21/Male34/Male 53/Female 22/Male64/Female 45/Male72/Female	Range of symptoms that include pain, limited jaw morbidity, swelling, dysphagia and odynophagia	Analysis of the synovial fluid, and radiographic imaging (MRI and CT scan)	Antibiotic therapy. Draining the arthrocentesis	The symptoms of the infected TMJ have improved. Other cases resulted in ultimate death or lasting complications
Nagarakanti SR et al., 2022	1	58/Male	Unstable mental state, discharge from the ear, cachexia	MRI and CT scan, microbiological testing	Surgical draining, IV nafcillin	Cardiac arrest mortality, hence the necessity of early detection and administering the ideal antibiotic therapy
Azmi et al., 2021	1 case	46 / F	Limited mouth opening for one month associated with pain and facial swelling.	Earlier diagnosed as internal derangement, but then later as TMJ	Orally administered ciprofloxacin Intravenous metronidazole and ceftazidime	Symptoms disappeared after administration of treatment and there were no recurrences reported even

Summary of TMJ septic arthritis cases referenced in this article

			Progressive swelling noted from left TMJ region to left infraorbital region. It was warm, tender and firm. Yellowish discharge oozed from the inner part of the cheek	septic arthritis	Left TMJ with arthrocentesis<br>Debridement of the wound and condylectomy on the left joint	six months after follow-up
Kito et al., 2024	1	33/M	Swelling, pain, problems with opening mouth, without malocclusion	Chronic infectious arthritis	Intravenous and oral antibiotics	The symptoms reduced after treatment administration
Symanski et al., 2023	Six cases	N/A	Suspected TMJ septic arthritis	Five diagnosed with septic arthritis and one with GVHD arthritis. The synovial fluid in the four of the five with	N/A	No sudden complications were recorded.

Summary of TMJ septic arthritis cases referenced in this article

				septic arthritis showed positive cultures		
Ângelo et al., 2023	1	68/M	Erythema, pre-auricular swelling, arthralgia in the right parts of the TMJ, and pre-auricular swelling	Septic arthritis of the TMJ and chronic suppurative otitis media	Targeted intravenous antibiotics for 30 days, arthroscopy on the right TMJ, the collection of joint fluid for analytical purposes, biopsy and lavage	Successful treatment administration with no recurrence after a 12-month period follow-up
Long et al., 2019	N/A	N/A	Warmth, erythema, pain in the joints, monoarticular arthritis, fever, limited mobility	Septic arthritis with a likely polyarticular involvement	Orthopedic surgery consultation, intravenous antibiotics and joint aspiration	Varied with the diagnostic promptness
